# Spinal Cord Infarction Presenting as Cauda Equina Syndrome With Concurrent Pulmonary Embolism: A Case Report

**DOI:** 10.7759/cureus.93778

**Published:** 2025-10-03

**Authors:** Aysha Rajeev, Mintu Mariam Baby, Saurav Krishnan

**Affiliations:** 1 Trauma and Orthopaedics, Gateshead Health Foundation NHS Trust, Gateshead, GBR; 2 General Medicine, Ulster Hospital, Belfast, GBR; 3 General Medicine, The Royal Victoria Infirmary, Newcastle, GBR

**Keywords:** cauda equina, cauda equina syndrome mimic, embolism, glp-1 receptor agonist, infarction, spinal cord, syndrome, vascular

## Abstract

Spinal cord infarction is rare and can be difficult to recognize early in emergency settings, especially when it mimics cauda equina syndrome (CES) with concurrent pulmonary embolism (PE). CES typically results from compression of lumbosacral roots, presenting with lower limb sensory changes or weakness and bladder dysfunction, whereas spinal cord ischaemia can present with overlapping features that delay diagnosis.

A 58-year-old woman presented with acute back pain, saddle numbness, urinary retention, and asymmetric leg weakness consistent with suspected CES. Initial lumbar MRI showed degenerative changes without compressive pathology. Within 48 hours, neurological status worsened to flaccid paraplegia with sphincter dysfunction. Repeat MRI demonstrated T2/STIR (short tau inversion recovery) signal from the conus to approximately T12, consistent with spinal cord infarction. Concurrently, she developed chest pain and hypoxia. CT pulmonary angiogram (CTPA) identified a saddle embolus in the main trunk of the pulmonary artery, and therapeutic anticoagulation was initiated. Workup revealed antiphospholipid antibody positivity. She was using oral hormone replacement therapy and a GLP-1 agent for obesity, constituting a prothrombotic context, although a direct arterial embolic source was not confirmed.

This case underscores three points: maintain a vascular differential for CES phenotypes with non-compressive MRI, consider early repeat MRI when symptoms evolve, and assess for systemic thromboembolism and prothrombotic risk factors to guide urgent management and follow-up.

## Introduction

Spinal cord infarction is uncommon, with an estimated incidence of approximately 12 per 100,000 and representing 6% of acute myelopathies and 1-2% of vascular neurologic pathologies [1,2]. Its clinical picture typically includes abrupt weakness or paralysis with bowel and bladder dysfunction, and early recognition can be challenging in emergency settings [3]. Reported precipitating contexts span thoraco‑abdominal aortic disease and surgery, embolic phenomena, dissection, systemic hypotension, spinal arteriovenous malformations, diving-related decompression illness, coagulopathies, cocaine use, and sickle cell disease [4-7].

Cauda equina syndrome (CES) most often arises from compressive lumbosacral pathology and presents with lower limb sensory changes or weakness, saddle anaesthesia, and bladder or bowel dysfunction, yet substantial overlap with the presentation of spinal cord ischaemia can delay diagnosis and alter early management [8]. Delayed recognition of spinal cord infarction can result in permanent, severe neurological disability, including persistent paralysis (from paraplegia to quadriplegia), loss of pain and temperature sensation, and autonomic dysfunction with neurogenic bladder and bowel, cardiovascular dysregulation, and sexual dysfunction. Early, accurate identification improves the chance to stabilise physiology, initiate anticoagulation when indicated, and begin intensive rehabilitation and supportive care, whereas greater initial injury severity is strongly associated with poorer long‑term functional outcomes. Diagnostic uncertainty is further compounded when the initial MRI of the lumbar spine shows degenerative changes without definitive compression, necessitating a high index of suspicion for vascular aetiologies and consideration of repeat or extended-segment imaging.

This report describes a 58‑year‑old woman who initially presented with a CES phenotype and subsequently evolved to spinal cord ischaemia in the setting of a prothrombotic context, highlighting the diagnostic pitfalls and the value of timely, serial MRI scans alongside risk‑factor appraisal.

## Case presentation

A 58-year-old woman presented to Accident & Emergency (A&E) with sudden-onset lower back pain while rising from the floor, accompanied by bilateral lower-limb paraesthesia and motor weakness, more pronounced on the right. She also reported saddle numbness and difficulty voiding. Past medical history included hypothyroidism, obesity, epilepsy, and depression. Regular medicines included oral hormone replacement therapy and a GLP‑1 agent for weight management.

On examination, she was alert and oriented with intact upper-limb function. Lower limbs showed normal tone, no clonus, and reduced power on the right. Babinski responses were equivocal, and deep tendon reflexes at the knees and ankles were absent. Hip flexion/extension 1/5, knee flexion/extension 2/5, ankle dorsiflexion/plantarflexion 1/5, toe dorsiflexion/plantarflexion 0/5, ankle eversion/inversion 0/5 (Medical Research Council (MRC) scale), with left-sided power 4/5 across muscle groups. Perianal sensation was altered, and a post-void bladder scan measured 600 mL, after which she was catheterised.

**Figure 1:**
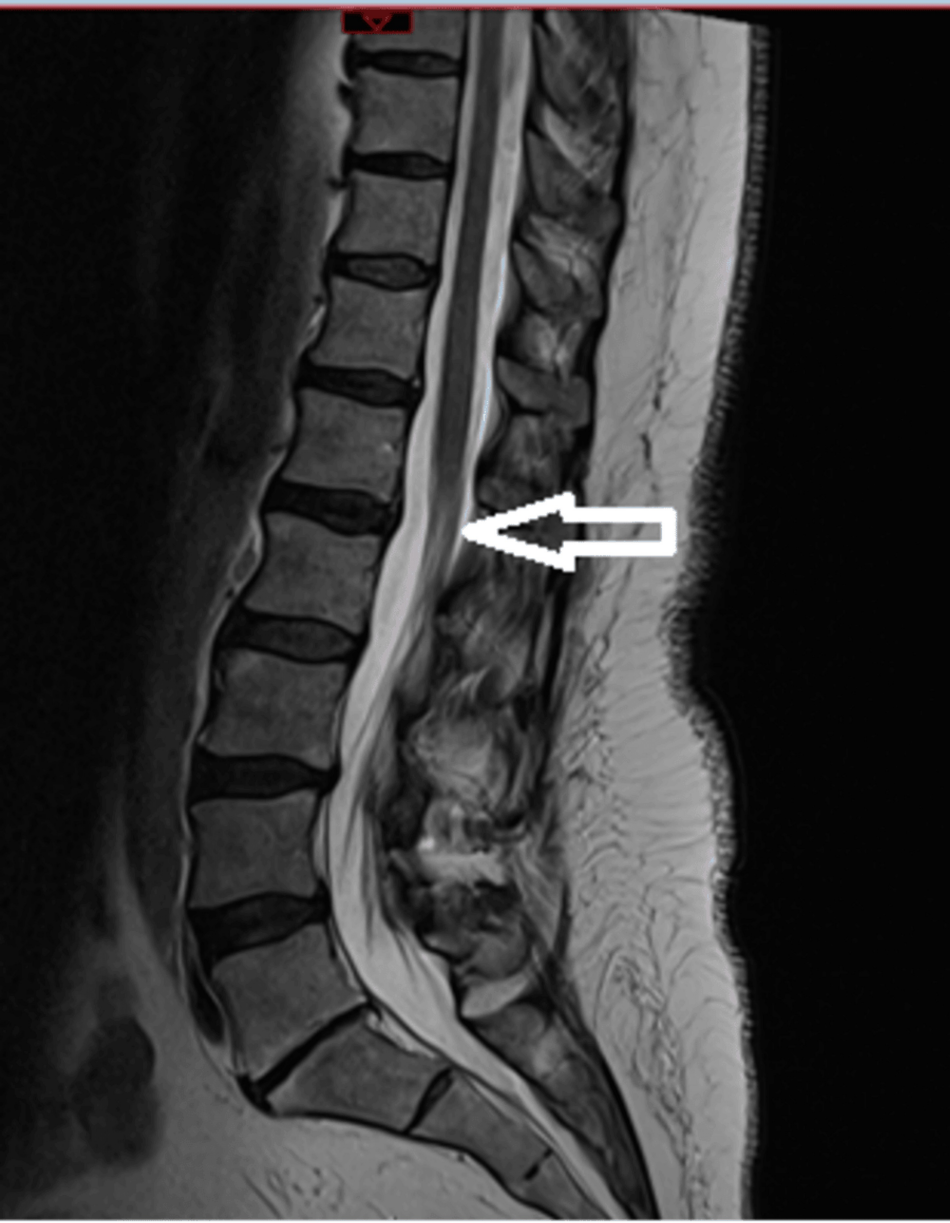
Sagittal T2‑weighted lumbar spine MRI demonstrating multilevel degenerative change without compressive cauda equina pathology No canal compromise or nerve‑root compression to account for cauda equina syndrome is identified.

A provisional diagnosis of cauda equina syndrome was made, and an urgent MRI was arranged. Lumbar MRI demonstrated L3-L4 and L4-L5 disc protrusions and L5-S1 degenerative change without compressive pathology consistent with CES (Figure 1). In accordance with the hospital protocol, the patient’s clinical findings and imaging were reviewed in consultation with the neurosurgical team. The recommendation was for conservative management with physiotherapy and analgesia. This approach was associated with transient improvement; the pain reduced, and a supervised trial without a catheter was successful, allowing the removal of the urinary catheter.

**Figure 2:**
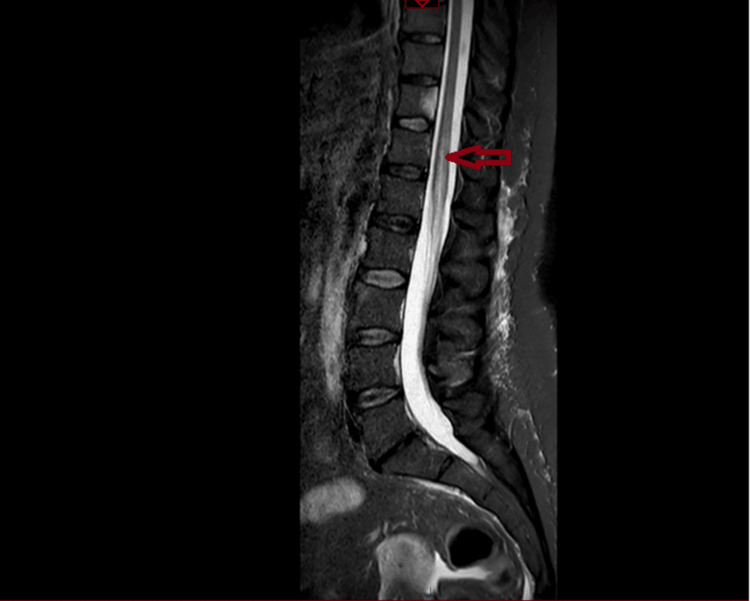
Sagittal STIR MRI of the thoracolumbar spine demonstrating linear intramedullary hyperintensity extending from the conus medullaris to approximately the T12 superior endplate, consistent with acute spinal cord infarction STIR: short tau inversion recovery

**Figure 3:**
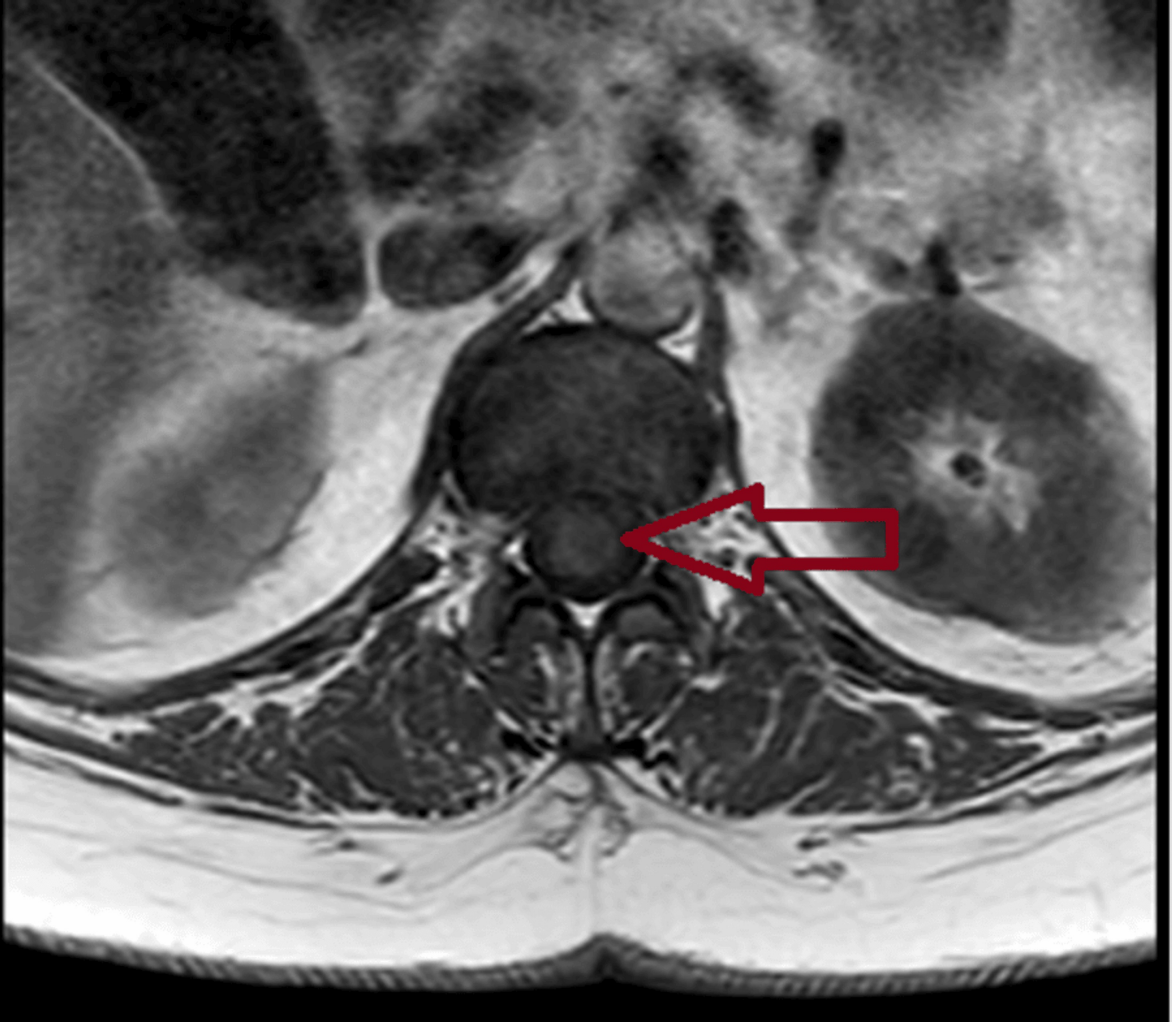
Axial T1‑weighted MRI at the conus level demonstrating intramedullary signal abnormality consistent with acute spinal cord infarction

Within 48 hours, neurological status deteriorated to 0/5 power in all lower-limb muscle groups bilaterally with areflexia, reduced sensation in all dermatomes, and urinary incontinence. Repeat MRI demonstrated T2/STIR signal change from the conus to the T12 superior endplate consistent with spinal cord infarction (Figures 2, 3). The repeat MRI demonstrated acute conus infarction, delineating the evolution from an initial CES‑like presentation on non-compressive imaging to established spinal cord ischaemia.

**Figure 4:**
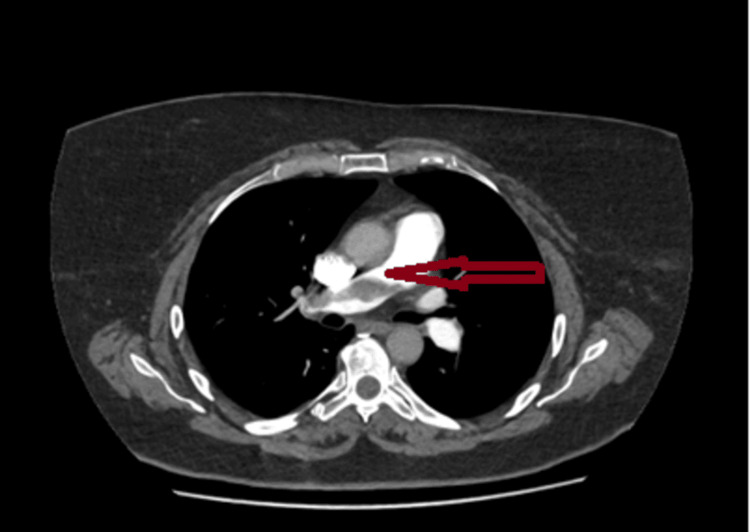
CT pulmonary angiography demonstrating a large saddle pulmonary embolus straddling the bifurcation of the main pulmonary artery with extension into both proximal pulmonary arteries, consistent with high embolic burden

She then developed acute chest tightness with hypotension, tachycardia, and hypoxia (oxygen saturation (SpO2) 92% on room air). The ECG demonstrated right bundle branch block (RBBB) with an S1Q3T3 pattern, lateral ST depression, and widespread T‑wave inversion--findings classically linked to acute PE and supportive in the clinical context, though not diagnostic on their own. CTPA confirmed a saddle pulmonary embolus (Figure 4). Therapeutic anticoagulation with tinzaparin was initiated, transitioning to oral anticoagulation. CSF culture was negative, MOG and aquaporin‑4 antibodies were negative, and antiphospholipid antibody testing was positive.

She was transferred to neurology for ongoing care and commenced intensive neuro‑rehabilitation. Her motor power gradually improved over the subsequent months, and at six months, she was able to mobilise with the assistance of two people, with continuing gains thereafter.

Table 1 presents the chronological clinical timeline of the patient.

**Table 1:** Chronological clinical timeline: presentation, radiology findings, and management SpO2: oxygen saturation; RBBB: right bundle branch block; PE: pulmonary embolism

Timepoint	Symptoms and signs	MRI findings	Key investigations	Interventions and course
Day 0 (ED presentation)	Sudden lower back pain while rising; bilateral lower‑limb paraesthesia; right‑predominant weakness; saddle numbness; urinary retention; knee/ankle reflexes absent; plantar responses equivocal	Lumbar MRI: L3–L4/L4–L5 disc protrusions; L5–S1 degeneration; no compressive cauda equina pathology (noncompressive study)	Post‑void residual 600 mL; catheterised	Analgesia; urinary catheter insertion; admission and monitoring
Days 1–2	Transient improvement in lower‑limb power; supervised trial without catheter (TWOC) to assess spontaneous voiding	—	—	Physiotherapy; observation during TWOC
Day 2 (neurological worsening)	Rapid progression to 0/5 power bilaterally; areflexia; reduced sensation across dermatomes; urinary incontinence	Repeat spine MRI: T2/STIR hyperintensity from conus to ~T12 superior endplate consistent with acute spinal cord infarction	—	Escalation of care; neurology input; rehabilitation planning
Day 2 (respiratory event)	Acute chest tightness; hypotension; tachycardia; hypoxia (SpO2 92% RA); ECG: RBBB, S1Q3T3, lateral ST depression, widespread T‑wave inversion (classic PE‑associated pattern, not diagnostic alone)	CTPA: large saddle pulmonary embolus	CSF culture negative; MOG/AQP4 negative; antiphospholipid antibodies positive	Therapeutic tinzaparin anticoagulation was initiated, then transitioned to oral anticoagulation; no BP augmentation was used as haemodynamics were normal
Months 1–6	Gradual neurological recovery with intensive rehabilitation	—	—	Intensive neuro‑rehabilitation; at 6 months, mobilising with the assistance of two; ongoing gains

## Discussion

Spinal cord infarction is rare but critical to recognise early because initial clinical features can overlap with compressive lumbosacral pathology [9], creating scope for a diagnostic delay when the first lumbar MRI shows only degenerative changes without compression. Conus medullaris involvement is uncommon overall, yet its presentation with acute back pain, saddle sensory disturbance, flaccid areflexia, and early sphincter dysfunction can closely mimic CES at onset. In this case, the transition from a CES phenotype with a negative initial lumbar MRI to a definitive conus-level T2/STIR signal on repeat imaging illustrates the importance of serial and extended-segment spinal MRI when neurology evolves despite noncompressive initial findings.

The vascular anatomy offers a pathophysiologic rationale for the observed pattern. The anterior spinal artery supplies the anterior two-thirds of the cord, with relative watershed vulnerability in the midthoracic to thoracolumbar region [3]; the artery of Adamkiewicz typically arises between T9 and L2 (left-sided in most) [3], and variability in radiculomedullary supply may predispose the conus to ischaemia under systemic or embolic stressors. A wide range of triggers and associations is reported, including aortic disease and repair, embolic disease, dissection, systemic hypotension, spinal arteriovenous malformations, decompression illness, coagulopathies, cocaine, and sickle cell disease; however, many cases remain idiopathic [10,11]. Imaging hallmarks of acute cord ischaemia typically include longitudinal T2 hyperintensity and STIR signal that may span multiple levels. Diffusion-weighted imaging can support the diagnosis but is frequently limited by technical constraints in the spine and may be normal early, warranting repeat MRI within 24-48 hours if suspicion remains high. The conus-to-T12 superior endplate signal in this patient aligns with an ischaemic aetiology and the clinical deterioration after a transient improvement phase.

The concomitant saddle pulmonary embolus demonstrated on CTPA during neurological worsening points to active venous thromboembolism (VTE) within a prothrombotic milieu, given oral hormone replacement therapy (HRT) and antiphospholipid antibody positivity. GLP‑1 exposure was present; however, evidence linking GLP‑1 receptor agonists to VTE is heterogeneous and inconclusive, so any contribution in this single case should be framed cautiously to avoid over‑attribution. 

A prothrombotic context was present, with three notable factors. First, the prolonged use of oral hormone replacement therapy confers an elevated risk of VTE, encompassing deep vein thrombosis and pulmonary embolism, with a higher risk observed for oral versus transdermal formulations [12]. Second, a GLP‑1 receptor agonist was being used for weight management. While GLP‑1 agents are established for type 2 diabetes and obesity, signals for VTE remain heterogeneous in the literature and should be interpreted cautiously in individual cases, notwithstanding reports suggesting increased risk in some analyses [13]. Third, antiphospholipid antibody positivity is associated with substantially increased VTE risk and supports a systemic prothrombotic milieu in this presentation [14].

Although a direct arterial source to the spinal circulation was not demonstrated, the coexistence of pulmonary embolism and these risk factors makes an embolic or thrombotic mechanism plausible for cord ischaemia, with transient episodes potentially explaining the initial CES‑like presentation before progression to established infarction on repeat imaging. The confirmed saddle pulmonary embolus on CTPA further corroborates active thromboembolism during neurological deterioration in this case.

Zalewski et al. have proposed diagnostic criteria and subtypes for spinal cord infarction, classifying cases as definite, probable, or possible ischaemia based on three core components: (1) clinical: rapid onset of severe neurological deficits, typically within 12 hours; (2) spinal MRI: demonstration of intramedullary hyperintense signal on T2-weighted images; (3) CSF analysis: absence of inflammatory findings. Our patient fulfilled both the clinical and imaging criteria, supporting a diagnosis of definite spontaneous spinal cord infarction [15].

In this case, the initial MRI scan was unremarkable. Given her relevant vascular risk factors, alternative diagnoses beyond cauda equina syndrome, such as evolving spinal cord infarction, should be considered early in the differential. Therefore, serial MRI scanning, together with cerebrospinal fluid analysis, represents the most appropriate strategy for the prompt identification of spinal cord infarction, particularly when standard MRI is initially negative and clinical progression persists.

Management of suspected spinal cord ischaemia prioritises optimising spinal cord perfusion, addressing reversible triggers, and initiating rehabilitation early. Where VTE is confirmed, therapeutic anticoagulation is indicated; in this case, tinzaparin followed by oral anticoagulation aligns with standard VTE care. Blood pressure augmentation and cerebrospinal fluid drainage are most established in peri-aortic surgical settings to improve spinal perfusion pressure. Outside these scenarios, they may be considered on a case-by-case basis within multidisciplinary teams. Neuroprotective strategies, such as hypothermia or thrombolysis, for primary spinal cord infarction remain investigational and should be discussed cautiously [16]. Intensive, multidisciplinary neurorehabilitation is essential. Functional gains over months, as observed here, are consistent with reported trajectories after incomplete cord ischaemia, although long-term outcomes can vary widely [17].

Practice implications arise for emergency, orthopaedic, and neurology services. First, maintain a vascular differential for CES-like presentations when the initial lumbar MRI lacks a compressive correlate, especially if deficits evolve or systemic thromboembolic features emerge. Second, repeat or extend MRI to include the thoracolumbar junction and conus early when neurology progresses, recognising that initial MRIs can be negative in acute ischaemia and become positive after 24-48 hours. When neurological deficits progress despite an initial noncompressive MRI, repeating spinal MRI within 24-48 hours is essential to detect evolving ischaemic changes and expedite management. Third, evaluate for systemic thrombosis and prothrombotic states, including VTE, antiphospholipid antibodies, and exogenous hormones, because identifying a prothrombotic context guides acute anticoagulation, secondary prevention, and follow-up planning. There is a clear need for prospective research to delineate vascular causes that present as CES mimics, including their frequency, imaging evolution, and evidence‑based protocols for timely recognition.

## Conclusions

Spinal cord infarction can closely mimic CES at presentation, particularly when acute back pain, saddle sensory disturbance, lower-limb weakness, and early bladder dysfunction coexist. A noncompressive initial lumbar MRI does not exclude a vascular aetiology. When neurology evolves, prompt repeat and extended-segment MRI, including the thoracolumbar junction and conus, can reveal emerging ischaemic changes that were initially occult, expediting diagnosis and management. The coexistence of pulmonary embolism and prothrombotic factors (oral HRT use and antiphospholipid antibodies) in this case underscores the need to screen for systemic thromboembolism and thrombophilia and to institute appropriate anticoagulation when VTE is confirmed. Early optimisation of spinal cord perfusion, careful consideration of context-specific measures, and initiation of intensive neuro‑rehabilitation are central to care, with functional gains possible over months despite severe initial deficits. Clinicians evaluating suspected CES should maintain a vascular differential, repeat imaging early when deficits progress, and appraise the thrombotic risk to guide acute treatment and secondary prevention.
